# Tat gets the "green" light on transcription initiation

**DOI:** 10.1186/1742-4690-2-69

**Published:** 2005-11-09

**Authors:** John Brady, Fatah Kashanchi

**Affiliations:** 1 National Cancer Institute, Laboratory of Cellular Oncology, Bethesda, MD 20892, USA; 2 The George Washington University School of Medicine, Department of Biochemistry and Molecular Biology, Washington, DC 20037, USA

## Abstract

Human immunodeficiency virus type 1 (HIV-1) Tat transactivation is an essential step in the viral life cycle. Over the past several years, it has become widely accepted that Tat exerts its transcriptional effect by binding the transactivation-responsive region (TAR) and enhancing transcriptional elongation. Consistent with this hypothesis, it has been shown that Tat promotes the binding of P-TEFb, a transcription elongation factor composed of cyclin T1 and cdk9, and the interaction of Tat with P-TEFb and TAR leads to hyperphosphorylation of the C-terminal domain (CTD) of RNA Pol II and increased processivity of RNA Pol II. A recent report, however, has generated renewed interest that Tat may also play a critical role in transcription complex (TC) assembly at the preinitiation step. Using in vivo chromatin immunoprecipitation assays, the authors reported that the HIV TC contains TBP but not TBP-associated factors. The stimulatory effect involved the direct interaction of Tat and P-TEFb and was evident at the earliest step of TC assembly, the TBP-TATA box interaction. In this article, we will review this data in context of earlier data which also support Tat's involvement in transcriptional complex assembly. Specifically, we will discuss experiments which demonstrated that Tat interacted with TBP and increased transcription initiation complex stability in cell free assays. We will also discuss studies which demonstrated that over expression of TBP alone was sufficient to obtain Tat activated transcription in vitro and in vivo. Finally, studies using self-cleaving ribozymes which suggested that Tat transactivation was not compatible with pausing of the RNA Pol II at the TAR site will be discussed.

## Tat transactivation: A historical perspective, initiation vs elongation

Transcription of the HIV-1 provirus is characterized by an early, Tat-independent and a late, Tat-dependent phase. Transcription from the HIV-1 LTR is increased several hundred-fold in the presence of Tat and the ability of Tat to activate transcription is essential for virus replication. Tat is an unusual transcription factor because it interacts with a *cis *acting RNA enhancer element, TAR, present at the 5' end of all viral transcripts (nt +1 to +59) [1-4]. In fact, TAR was the first demonstration of a RNA enhancer element. Unlike other eukaryotic enhancers, however, the TAR element was only functional when it was placed 3' to the HIV promoter and in the correct orientation and position [5]. The location of the TAR in transcribed regions was surprising, and to many, inconsistent with a role for TAR in transcription initiation. In fact, the uniqueness of the RNA enhancer element drove many investigators to search for unique pathways in HIV Tat transactivation. When Kao et al. [6] reported that in the absence of Tat the majority of RNA polymerases initiating transcription stall near the promoter, and later Laspia et al. [7] reported a small effect of Tat on transcription initiation but a large effect on transcription elongation, the initiation model quickly lost support. The observation that Tat binds specifically to the TAR RNA [8] and could function as an RNA binding protein [9] gave further support for the elongation model, and it became quite well accepted that through interaction with TAR, Tat promotes the assembly of an active transcription elongation complex. The more recent finding that Tat promotes the binding of P-TEFb, a transcription elongation factor composed of cyclin T1 and cdk9 [10] and, more recently, Brd4 in the active nuclear complex [11] seemed consistent with the elongation model. In fact, it has been shown that the interaction of Tat with P-TEFb and TAR leads to hyperphosphorylation of the C-terminal domain (CTD) of RNA Pol II and increased processivity of RNA Pol II [12-22]. Moreover, Tat induces P-TEFb dependent phosphorylation of Tat-SF1 and SPT5 [23]. While TAR plays a critical role in Tat transactivation, it is also clear that optimal Tat transactivation of HIV-1 gene expression requires upstream transcription co-factors. Along these lines, it has been reported that Tat physically interacts with the pre-initiation complex including transcription factors such as Sp1 [24], TATA binding protein (TBP) [25-27], cylinE/cdk2 [28], TFIIH [21,22], Tip60 [29], RNA Pol II [30,31], as well as coactivators such as CBP/p300 [32] and p/CAF [33,34]. Several excellent reviews of the role of Tat in transactivation have been published [1,35-40].

## A role for Tat in transcription preinitiation complex assembly

A recent report from M. Green's lab has, however, generated renewed interest that Tat's primary effect may in fact be at the transcription complex (TC) assembly stage at the pre-initiation step upstream of the +1 area, thereby promoting both transcription initiation and elongation of HIV-1 promoter [41]. The authors reported that Tat stimulates TC assembly through a TAF-less TBP complex, thereby promoting initiation and elongation [41]. The stimulatory effect was evident at the earliest step of TC assembly, the TBP-TATA box interaction. Furthermore, much like the scenario in yeast, transcription of protein-coding genes may involve alternative TCs that differ by the presence or absence of certain TAFs. To analyze transcription stimulation by Tat and other activators, such as VP16 and E1A, they performed ChIP experiments in transiently transfected mammalian cells. Following addition of Tat, there was a large increase in association of TBP, TFIIB, mediator (enabling transcriptional activators to regulate transcription by RNA polymerase II), and RNA polymerase II with the promoter. The increased binding of these basal transcription factors paralleled the increase in transcription. Interestingly, although TBP and the other GTFs were efficiently recruited to the promoter in the presence of Tat, there was no significant recruitment of the two TAFs analyzed, TAF1 (TAFII250) or TAF5 (TAFII100). Consistent with their transfection data, they observed the presence of TBP, TFIIB, mediator, Sp1, P-TEFb and RNA polymerase II with the integrated proviral promoter in chronically HIV-1-infected cell lines, 8E5/LAV and U1.

In parallel control ChIP experiments analyzing Gal4-VP16 and Gal4-E1a, the investigators demonstrated that these activators supported assembly of a transcription complex that contained all of the GTFs, including TAFII250 and TAFII100. By contrast, Gal4-Tat directed assembly of a TC in which the TAFs were present at levels significantly below that of TBP and other GTFs. Remarkably, when assaying for effect of cyclin T1 and CDK9, they observed that P-TEFb was responsible for recruitment of this unique TBP complex.

Finally, they concluded that RNA polymerase II was not detected either near or far downstream of the transcription start site in the absence of Tat and thus provided no evidence for a paused (or stalled) RNA polymerase II. Consistent with their ChIP data, nuclear run-off experiments showed that Tat increased the density of RNA polymerase II 9- to 15-fold within the first 25 nucleotides downstream of the transcription start site, indicating that Tat stimulates initiation.

It is interesting to note that while the authors do not see a dependency on TAFII250 for Tat transactivation on the HIV LTR, the interaction of Tat and TAFII250 is important for Tat-mediated transcription repression. Tat represses transcription of both the MHC class I genes and the beta2-microglobulin gene. Repression results from the interaction of Tat with the TAF1 component of the general transcription factor, TFIID and depends exclusively on the C-terminal domain of Tat, beginning at amino acid 73, with a C-terminal limit between amino acids 80 and 83. Tat repressor function also depends on the presence of a lysine at position 41, located within the core of the protein. Tat repressor activity is independent of two N-terminal domains essential for transactivation: the acidic segment and the cysteine-rich region. The C-terminal domain of Tat binds to a site on TAF1 that overlaps the acetyl transferase (AT) domain, inhibiting TAF1 acetyl transferase (AT) activity. Furthermore, promoters repressed by Tat, including the MHC class I promoter, are dependent on TAF1 whereas those that are not repressed by Tat, such as SV40 and MuLV promoters, are independent of functional TAF1 [42-45].

## Further evidence for the role of Tat in preinitiation complex assembly

While these studies have renewed interest in the role of Tat in promoting transcription initiation, the idea is certainly not new. For instance, Kashanchi et al. reported in 1994 that the transcriptional activity of HeLa extracts were depleted after chromatography on a Tat affinity column, through specific retention of TBP and some TAFs. The core domain of Tat, amino acids 36–50, was required for the interaction of Tat with TBP and a mutation at Lys 41, which abolishes transactivation, abolished interaction with TBP [46,47]. In fact, based on these results and earlier studies that Tat increased transcription initiation complex stability in cell free assays [48], the authors speculated in this paper that Tat may increase the association or dissociation of TFIID, or recruit a particular species of functionally different TFIID to the HIV template. In contrast to the studies of Raha et al. [41], by western blot analysis Kashanchi et al. [46] detected TAFII250 in the Tat-induced transcription complex. The relative abundance of TBP and TAFII250 was not quantitatively evaluated, however, so it is possible that less TAFII250 was associated with the Tat-TBP complex.

Other studies have also pointed toward the functional interaction of Tat and TBP. The activity of Tat, either wild-type or fused to the DNA binding domain of GAL4 (GBTat), was tested using reporter constructs containing GAL4 binding sites upstream of a minimal promoter corresponding to the HIV-1 TATA box, with or without the TAR element. Overexpression of TBP led to a dramatic increase in the activity of the GBTat protein. Analysis of several Tat mutants indicated that both the cysteine-rich and the core domains of this transactivator were necessary and sufficient to activate transcription when TBP was overexpressed. *In vitro *experiments showed that Tat binds specifically to TBP, and follow up *in vivo *experiments indicated a correlation between the ability of different Tat mutants to bind TBP and their capacity to activate transcription *in vivo *[27,49-51]. Still other studies that looked at the interaction of TBP and Tat concluded that activation of the LTR requires steps in addition to TBP recruitment [52].

The Hernandez lab has previously shown that TBP bound to the TATA box was required for the synthesis of short and full-length transcripts as well as for Tat activation and that both yeast TBP and the carboxy-terminal domain of human TBP could replace full-length human TBP for these processes [53]. Similar studies from the Lania lab indicated similar activation by a TBP fusion. For instance, to determine the synergy between Tat and GAL4-TBP in the absence of any DNA-bound activator, the G1-38HIV reporter was transfected into HeLa cells with the GAL4-hTBP and a Tat expression vector. Tat alone had no effect on transcription, however, co-expression of Tat strongly stimulated GAL4-hTBP transcription in the absence of any DNA-bound activator. Synergy between Tat and DNA-bound TBP protein was further confirmed by analysis of the levels of specific transcripts, which were determined by RNase protection assay [54]. Therefore, artificial recruitment of human TBP to the enhancerless HIV minimal promoter was found to trigger gene expression, and coexpression of Tat resulted in a marked synergy. The functional cooperation between TBP and Tat was further demonstrated using the Drosophila Schneider SL2 cells [55].

Finally, with regard to the functional significance of Tat's role in transcription complex assembly and levels of nonprocessive transcription from the HIV-1 LTR, two manuscripts from the Jeang lab are worth noting. First, a central question was asked in whether the LTR promoter "presynthesizes" short nascent TAR RNA-containing transcripts that remain poised awaiting Tat. To address this question, the investigators used a self-cleaving ribozyme to define a time window during which Tat action occurred, which measured Tat trans-activation against two biological processes: RNA chain elongation and RNA self-cleavage [56]. To do this, they placed a rapidly self-cleaving ribozyme downstream of TAR. The experimental model assumed that if the ribozyme self-cleavage reaction was sufficiently rapid then it should sever the TAR-Tat complex that was attached to the nascent RNA chain and thus prevent an interaction with the LTR promoter. Therefore, the speed of one process (trans-activation) was compared against another (RNA chain elongation leading to self-cleavage). From their experiments, they concluded that an accumulation of paused TAR transcripts between +42 and +80 was unlikely, and the evidence that rapid cleavage at +80 did affect (rate determine) the overall trans-activation process was not compatible with pausing at this location on the DNA template. Control experiments demonstrated that the observed reduction in expression was specific for a functional ribozyme and specific for trans-activation (as opposed to a perturbation in basal activity or in RNA stability).

Second, when examining the short (S) and long (L) form of HIV-1 RNA in an integrated provirus setting *in vivo*, they suggested that S RNAs, while seen in unintegrated DNA and/or cell-free assays, were not prevalent in the context of integrated proviruses [57]. Basal transcription from a vector containing SV40 sequence (pHIVCATSV) in Cos cells was characterized by an abundance of S transcripts, while a normal HIV vector (pLTRCAT) produced no such RNAs. With Tat, both plasmids transcribed comparable amounts of L transcripts. They concluded that abortive transcripts may simply reflect transcription that occurs as a consequence of replication induced by T antigen in cell lines tested. These data were also consistent with earlier reports on Tat's effect of TC complex stability when using cell-free assays [48].

While focus of the studies on Tat function was heavily placed on the role of cellular kinases, protein phosphatases might also play an important role in the early stages of HIV-1 transcription. Ammosova et al. [58] have shown that PP1 and PP2A dephosphorylate CDK9 and that inhibition of PP1 or PP2A phosphatase activity decreases HIV transcription in vitro and in vivo. While the authors concentrated on the activity of PP1 and PP2A on autophosphorylation sites, which include the activation site at Thr 186 and more C-terminal phosphorylation sites, it is possible that these phosphatases play a role in removing inhibitory CDK9 phosphorylation sites in the preinitiation complex [[23], M. Zhou, personal communication].

## Chromatin structure

In considering the effect of Tat on transcription initiation and elongation, the effect of chromatin structure on the integrated genome must be considered. Investigators have shown that chromatin exerts a strong repressive role on transcription initiation. Interestingly, in a 2003 study using chronically infected U1 cells treated with phorbol ester, Lusic et al. reported that Tat promotes the specific recruitment of histone acetyltransferases to the viral promoter, facilitating acetylation of histones H3 and H4 at distinct nucleosomal regions, before the onset of viral mRNA transcription [59]. In a separate study, Kiefer et al. reported that nucleosome remodeling, not histone acetylation, is the limiting step in transcriptional activation in U1 promonocytes [60]. It is possible therefore, that Tat facilitates chromatin modifications, assembly of the initiation complex and transcription elongation in a series of sequential, coordinated events that leads to high levels of HIV transcription.

## Similarities to viral transactivators Herpesvirus VP16 and IE, Adenovirus E1A and SV40 T-antigen and HTLV-1 Tax

We should not be surprised by the complexity of the Tat transactivation process and the multifaceted effect of Tat on multiple transcription factors involved in LTR regulation. Examination of viral activators and their mechanism of activation indicate how small DNA or RNA viruses have evolved intricate mechanisms for controlling viral and cellular gene expression. For example, the Herpesvirus VP16 activation domain can be divided into two modules – an N-terminal subdomain (VPN) and a C-terminal subdomain (VPC). It has been shown [61] that VPC stimulates core promoters that are either independent of or dependent on TAFs (TATA box Binding Protein-Associated Factors). In contrast, VPN only activates the TAF-independent core promoter, and this activity increases in a synergistic fashion when VPN is dimerized (VPN2). The VPN subdomain of VP16 also facilitates assembly of a transcriptional complex containing TBP: TFIIA:TFIIB, which lacks TAFs, and provides a mechanism that could function at TAF-independent promoters. Thus, the viral activator facilitates transcription through multiple functional pathways.

Along the same lines, biochemical and genetic evidence has suggested that the Herpesvirus IE proteins may perform functions similar to those of the TAFs in the transcriptional complex. The IE proteins expressed from the intact major IE gene, and to a lesser extent IEP86 alone, could rescue the temperature-sensitive (*ts*) transcriptional defect in TAFII250 BHK-21 ts13 cell line [62].

The adenovirus E1A protein is also a well studied transcription activator. The 48-amino-acid conserved region 3 (CR3) of E1A, which is responsible for mediating transactivation, appears to target several proteins of the transcription initiation complex, including ATF-2, and components of the basal transcription factor TFIID, including TBP, hTAFII250, hTAFII55, and hTAFII135 [63]. This interaction allows E1A to stabilize the TFIID-TFIIA complex to increase the level of activated transcription *in vivo*.

Another viral activator, SV40 large T-antigen has also been shown to specifically enhance the formation of the TBP-TFIIA complex on the TATA element. The ability to facilitate TBP/TFIIA binding was complex and promoter dependent since T-antigen could activate simple promoters containing the TATA elements from the *hsp70 *and c-*fos *gene promoters but failed to significantly activate similar promoters containing the TATA elements from the promoters of the SV40 early and adenovirus E2a genes. Furthermore, the ability to stabilize the TBP-TFIIA complex on the *hsp70 *and c-*fos *TATA elements, and not on the SV40 early and E2A TATA elements, correlated with the ability or inability to activate promoters containing these TATA elements [64]. Interestingly, in the ts13 cell line, T-antigen could rescue the temperature-sensitive (ts) defect in TAFII250. In contrast, neither E1A, small t-antigen, nor mutants of T-antigen defective in transcriptional activation were able to rescue the ts defect [65], further implying that T-antigen may act like a TAF activator.

Finally, while investigating the effect of HTLV-1 Tax on the pre-initiation complex assembly, it has been shown that Tax facilitates the binding of a variety of transcription factors including CREB, TFIIA, CBP/p300 and PCAF [66-69]. Interestingly, Caron et al., have shown that transactivation by Tax was correlated with its ability to interact with the C-terminal moiety of the TBP and hTAFII28 in transfected HeLa cells [70]. An increase in the intracellular concentration of hTAFII28 augmented transactivation by Tax. This effect was also seen in COS-7 cells that have low levels of endogenous TAFII28. TBP and hTAFII28 also cooperated to allow Tax activation of the entire HTLV-I promoter and to partially rescue the phenotype of Tax mutants that had an impaired ability to activate transcription. The authors speculated that since TBP was present in all three cellular RNA polymerases, an increase in the concentration of hTAFII28, which binds directly to TBP, may compete with the TAFIs and TAFIIIs and drive more TBP into the formation of a TFIID complex interacting with Tax. According to this model, overexpression of both TBP and hTAFII28 would most efficiently raise the concentration of TFIID complexes capable of functioning with Tax.

## Future considerations

Recent technical advances, such as ChIP and siRNA assays, allowed Raha et al. [41] to more clearly demonstrate that Tat facilitates TC assembly at the HIV initiation site in vivo. The results of this study are consistent with and supported by previous studies which demonstrated that Tat, pTEFb and HATs are present on the HIV promoter and support a role of Tat in transcriptional initiation [23,32,59,71]. It should be noted that, in addition to technical advances, the ability to detect Tat in transcription initiation is likely dependent upon the experimental system. Along these lines, it should be noted that other recent ChIP data are more consistent with an effect of Tat at transcription elongation. Bres et al. recently reported that in HeLa P4 cells SPT5, SPT6, RNAP II and Ser 5-P CTD were present on the integrated HIV promoter in the absence of Tat and ongoing transcription [72]. Future work will continue on the exciting and multifaceted and perhap sequential role of Tat in chromatin remodeling, preinitiation complex assembly, elongation, and processing and will include questions such as: 1) How P-TEFb, which was initially discovered as an elongation factor, selectively recruits TBP alone or in complex with other TAFs and activators; 2) Are there TBP associated complexes that are selective to Tat and not to other cellular promoters, and can they be purified to homogeneity; 3) Is the TBP recruited from the PolI or PolIII TC; 4) Does Tat act similarly to TAF subunits replacing some or all of the TFIID TAF subunits *in vivo*; 5) Can the data be reproduced in primary T- and monocytic latent patient samples? One thing is certain however. More research and funding is needed to define various mechanisms of Tat function in the hope that the data will result in finding the very first specific HIV transcription inhibitor *in vivo*.

**Figure 1:**
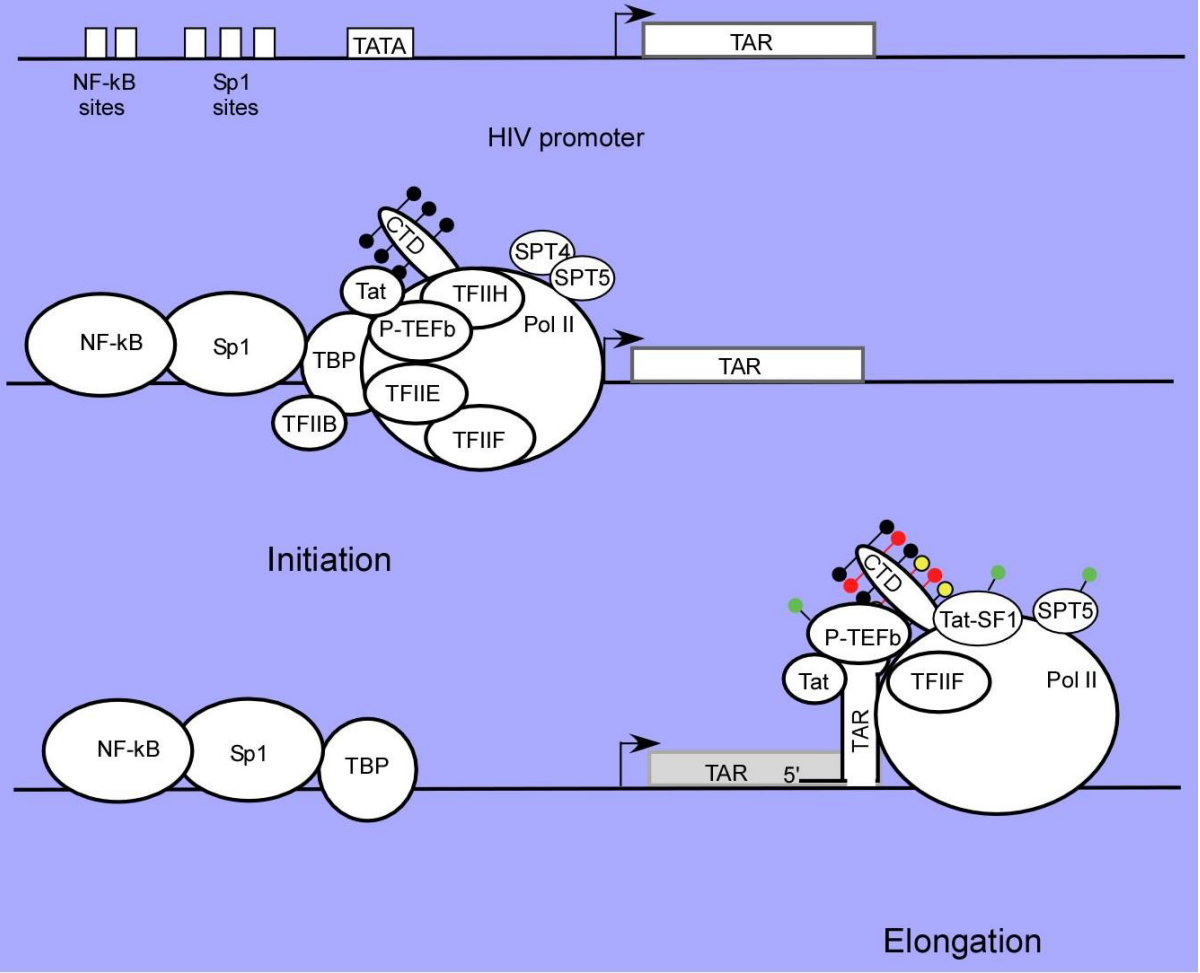
The HIV promoter is comprised of a series of transcription control elements including NF-kB, Sp1, TATA box, RNA initiation site and the downstream TAR RNA enhancer element. In the presence of Tat, a complex interaction between activators which include NF-kB and/or Sp1 bind to the upstream control region and interact with transcription factors which include, but may not be limited to, TBP, TFIIH, P-TEFb and RNA Pol II. Data from several laboratories now support a role for Tat in transcription complex assembly. Tat and P-TEFb facilitate the binding of TBP to the complex, setting the stage for binding of other basal transcription factors and assembly of the preinitiation complex. In the initiation complex, although both TFIIH and P-TEFb are present, the Pol II CTD is phosphorylated primarily by TFIIH at Ser5 (black). Following synthesis of the TAR RNA enhancer and loss of TFIIH from the elongation complex, P-TEFb is autophosphorylated at Thr186. Transcription elongation requires the interaction of Tat and P-TEFb with the TAR RNA which facilitates the phosphorylation of the Pol II CTD at Ser2 (red) and Ser5 (yellow), as well as the phosphorylation of Tat cofactors Tat-SF1 and SPT5. Whether the Tat and P-TEFb bound to TAR are transferred from the initiation complex, or represent the binding of additional Tat and P-TEFb remains to be established. The Tat-modified kinase activity of P-TEFb is preferentially sensitive to low concentrations of DRB or flavopiridol. This model assumes that the Tat and P-TEFb associated with the initiation complex transfers to the TAR RNA enhancer and perhaps to the elongation complex, a point that has not yet been demonstrated.
